# Reduction in inappropriate hospital use based on analysis of the causes

**DOI:** 10.1186/1472-6963-12-361

**Published:** 2012-10-17

**Authors:** Víctor Soria-Aledo, Andrés Carrillo-Alcaraz, Benito Flores-Pastor, Alfredo Moreno-Egea, Milagros Carrasco-Prats, José Luis Aguayo-Albasini

**Affiliations:** 1 Hospital Training and Research Unit; Morales Meseguer Hospital, Murcia, Spain; 2 General Surgery Department, Morales Meseguer Hospital, Murcia, Spain; 3 General Surgery Department, Santa Lucía Hospital, Cartagena, Spain; 4 Hospital General Universitario “J.M. Morales Meseguer” Calle, Marqués de los Vélez, Murcia, 30008, Spain

**Keywords:** AEP, Inadequacy, Inappropriate stay, Hospital stay, Hospital cost, Inappropriate admission

## Abstract

**Background:**

To reduce inappropriate admissions and stays with the application of an improvement cycle in patients admitted to a University Hospital. The secondary objective is to analyze the hospital cost saved by reducing inadequacy after the implementation of measures proposed by the group for improvement.

**Methods:**

Pre- and post-analysis of a sample of clinical histories studied retrospectively, in which the Appropriateness Evaluation Protocol (AEP) was applied to a representative hospital sample of 1350 clinical histories in two phases. In the first phase the AEP was applied retrospectively to 725 admissions and 1350 stays. The factors associated with inappropriateness were analysed together with the causes, and specific measures were implemented in a bid to reduce inappropriateness. In the second phase the AEP was reapplied to a similar group of clinical histories and the results of the two groups were compared. The cost of inappropriate stays was calculated by cost accounting. Setting: General University Hospital with 426 beds serving a population of 320,000 inhabitants in the centre of Murcia, a city in south-eastern Spain.

**Results:**

Inappropriate admissions were reduced significantly: 7.4% in the control group and 3.2% in the intervention group. Likewise, inappropriate stays decreased significantly from 24.6% to 10.4%. The cost of inappropriateness in the study sample fell from 147,044 euros to 66,642 euros. The causes of inappropriateness for which corrective measures were adopted were those that showed the most significant decrease.

**Conclusions:**

It is possible to reduce inadequacy by applying measures based on prior analysis of the situation in each hospital.

## Background

Inappropriate hospital use has been defined as a hospital admission to provide care that could have been given in a less complex health-care environment and at a lower cost [1,2].

Among the instruments for objective evaluation designed to determine the rate of appropriate stays the most well-known and widely used is the Appropriateness Evaluation Protocol (AEP), developed in the late seventies by Gertman and Restuccia [3] and reviewed in the eighties [4], and which has been validated in a number of studies [5-9]. It has proved a useful tool for identifying inappropriate stays and admissions of patients in acute care hospitals and the causes that determine them. Other methods, such as the Deley Tool developed by Selker, have proved useful for classifying the causes of inappropriate stays as identified by the AEP, although it is less commonly used.

The AEP has had various applications, corresponding usually to inappropriateness studies in specific clinical services such as emergencies and medical and surgical services [10-17]. Only a few studies have conducted a global inappropriateness study on an acute care hospital as a whole [18,19].

It has also been used to estimate the increase in costs associated with nosocomial infections [20,21]. Other not directly accountable benefits of a hospital inappropriateness study are: 1) better health planning, since objective criteria are introduced that allow evaluation of requests for an increase in resources, construction of new centers or the creation of new services; 2) identification of patient groups with a high risk of inappropriate use; and 3) attribution to each of the echelons of the health system their share of the responsibility for inappropriate use.

To learn the financial repercussions of inappropriateness hospitals must incorporate changes into their financing systems by moving them progressively towards a system of activity budgeting based on case mix, for which the cost per process must be known.

Most of those who have used the AEP to evaluate appropriate admissions or stays have done so for descriptive studies, but few have used the data to implement improvements [22]. The use of feedback to physicians with a view to reducing inappropriate hospital use has been contrasted by various authors [23-26], who show that the feedback strategy has been effective for reducing both inappropriate stays and total length of stay. However, the results reported by others who use feedback alone are not entirely satisfactory [26].

The AEP is a review instrument designed to determine the reasons identifying inappropriate use of a bed-day, but with the aim of building doctors’ awareness and promoting action and decision-making, without which any review is pointless.

The main objective: To reduce inappropriate admissions and stays with the application of an improvement cycle in patients admitted to a University Hospital. The secondary objective is to analyze the hospital cost saved by reducing inadequacy after the implementation of measures proposed by the group for improvement.

## Method

The J.M. Morales Meseguer Hospital in Murcia is a General University Hospital for adults belonging to the Murcia Health Service, which is financed entirely from public funds. It serves a population of 320,000 inhabitants and has 426 beds for conventional stays, 28 Surgical Day Hospital beds and 15 Medical Day Hospital beds. The mean rate of occupancy in 2005 was 84.5% ± 6.6%.

### Study design

A retrospective pre- and post-intervention study, was conducted to assess the improvement of inappropriate admissions and stays in our hospital after implementation of a group of measures. In the first phase (pre) we used a retrospective audit to study a randomized sample of the medical records of patients admitted to the hospital in 2005 (control group) using the AEP [27]. In a second phase an improvement cycle was performed by implementing measures to reduce inappropriate admissions and stays. Finally, in a posterior phase (post) we carried out a second evaluation by re-measuring the inappropriate admissions and stays in a retrospective randomized sample of medical records in 2007 (intervention group).

Excluded were patients admitted to ICU, Reanimation, Medical Day Hospital, Surgical Day Hospital and those with a stay of less than 2 days. The sample size for inappropriate stays was determined on the assumption that if the rate of inappropriate stays was 30%, and with the adoption of intervention measures, there would be a reduction of 10%, accepting an alpha risk of 0.05 and a beta risk of 0.10 in a bilateral contrast. Thus a total of 1450 subjects (725 in the control group and 725 in the intervention group) were required to detect the estimated difference. To study inappropriate stays we analyzed two days from the whole hospital stay chosen at simple random from each of the histories to assess the appropriateness of admission. The study presented has been approved by the ethics committee of clinical trials of the Hospital JM. Morales Meseguer.

The variables studied are shown in Table 1. Holiday periods were considered from 16 July to 15 September and the fortnights corresponding to Easter and Christmas. Outlying patients were defined as those admitted outside their corresponding Nursing Unit. Inappropriate admission is considered when on the day of admission the patient does not meet any criteria for hospitalization according to the AEP. Inappropriate stay is considered when on the selected day none of the hospitalization criteria according to the AEP are fulfilled.

**Table 1:** Variables studied

**1**	**Number of Clinical Records**
**2**	**Age.** Quantitative continuous variable, expressed in years.
**3**	**Sex.** Categorical variable.
**4**	**Date of admission.** This variable studies the month and day of the week of both admission and stays: Quantitative ordered variables.
**5**	**Admission in holiday period.** Qualitative variable. This variable considers an admission or stay during the summer holidays from July to September, Easter and the Spring Festival week, as well as the Christmas week and New Year.
**6**	**Days of total stay in Hospital.** Quantitative continuous variable.
**7**	**Responsible clinical speciality:** medical or surgical service responsible for the patient. Qualitative variable. These are grouped into Internal Medicine, Cardiology, Infectious Diseases, Haematology, Other Medical, General Surgery, Traumatology and Other Surgical.
**8**	**Diagnosis.** Refers to diagnosis on discharge of the patient coded according to the international standards of the ICD-9-CM Classification and DRG. Qualitative variable.
**9**	**Type of admission.** Categorical variable: programmed or emergency.
**10**	**Origin of admission.** Qualitative variable: emergencies, consultation, home or other hospital.
**11**	**Hospital occupancy rate on day of admission studied.** Quantitative continuous variable.
**12**	**Hospital occupancy rate on day of stay studied.** Quantitative continuous variable.
**13**	**Number of patients admitted on day of admission studied.** Quantitative continuous variable.
**14**	**Nursing Unit where patient is located.** Indicates whether the patient is in the corresponding ward or not (“outlying patient”). Qualitative variable.
**15**	**Criteria met for appropriateness of admission,** according to the AEP classification of reasons.
**16**	**Criteria met for appropriateness of stay,** according to the AEP classification of reasons. Cause of inappropriateness of admission when the admission is inappropriate.
**17**	**Cause of inappropriateness of stay** when the stay is inappropriate.
**18**	**Cause of inappropriateness of admission** when the admission in inappropriate.

One thousand four hundred and fifty clinical histories were selected proportionally to the number of admissions per medical service. The inappropriate admission protocol was applied to the 1450 histories and the inappropriate stay protocol to 2675 stays.

After analyzing the data observed in the control group in 2005 and the results of other published papers, representatives from the Medical Services, Hospital Management and Work group proposed a series of measures to reduce inappropriate hospital use: 1. Information to physicians in clinical services that decide on hospital admissions and stays. Educational sessions were held with all the clinical services to report the results and discuss possible measures that might reduce inappropriateness in each service. The working group shared the results of the first evaluation in each clinical service, with particular focus on the services with the highest percentage of inadequacy. In some cases, the retro-information had to be repeated when the team found that a good level of cooperation from doctors had been attained. Laminated leaflets were also distributed recalling the criteria for admission and accommodation included in the AEP; 2. Diffusion of the use of a discharge pre-report. In addition to remembering the demonstrated effectiveness of this measure in other studies, Hospital Management sent briefing notes to the various clinical services recommending the use of a pre-report; 3. Creation of a circuit of special outpatient radiological studies in the emergency department and for patients recently diagnosed or suspected with oncological disease. Patients considered a priority from emergencies and those recently diagnosed with cancer were scheduled on the same preferential list as those admitted to the hospital; 4. Relocation of “outlying” patients every evening by the duty nursing supervisor when beds were available for it.

**Figure 1:**
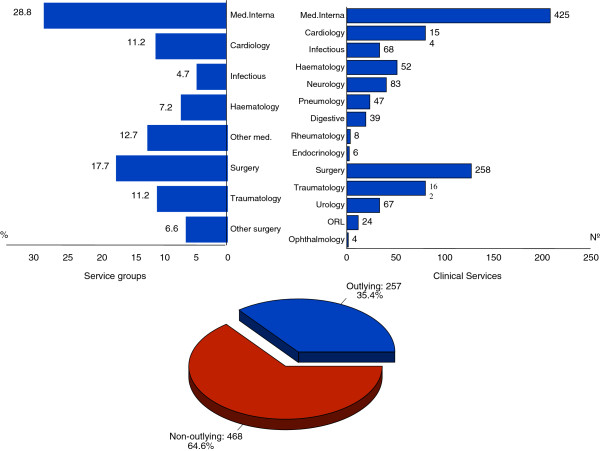
Service and location of patient admission.

The study sample, from both clinical histories and stays for analysis, was obtained by simple randomness via computer generation of a table of pseudorandom numbers. The sample was taken from the hospital’s clinical history archive. The selected number of histories for study was pondered for each of the services. In 2005, 10,534 patients were admitted to the Morales Meseguer Hospital with a stay of two or more days. The distribution of clinical histories by department is shown in Figure 1.

### The AEP

To identify inappropriate use we used the version in Spanish modified with permission from the Avedis Donabedian Foundation.

Three assessors were selected to apply the AEP: an internal resident doctor and two ward nurses with experience in the management of clinical histories on paper and on computer. By way of training the assessors were given a theoretical and practical course on the use of the AEP.

A concordance study was carried out among the three reviewers with two categories (appropriate and inappropriate). The sample size was calculated by assuming an inter-observer agreement of 80% and an accuracy of 5%. Thus we assessed 37 clinical histories to which the admission protocol was applied and 37 primaries stays and 31 secondary stays to which the appropriate stay protocol was applied [27].

### Statistical analysis

The quantitative variables are expressed as a mean ± standard deviation and the qualitative variables as percentages. The comparison between the rate of inappropriate admissions and stays in the pre- and post-groups was performed using the application of the Pearson Chi2 test or Fisher exact test. The identification of risk factors for inappropriateness was performed by unvaried analysis using the Chi 2 test or the Fisher exact test if the variables were qualitative and by the Student t test or single-factor ANOVA if the variables were quantitative. All the analyses were done with bilateral contrast and the p value was considered significant if less than 0.05. The variables that in the unvaried analysis showed a significance level of less than 0.05 were introduced into a multivariate model using logistic regression to analyze independent risk factors for admission and inappropriate stay. The software packages used were Granmo version 5.0 for Windows, EPIDAT 3.1 for Windows and SPSS 15.0 for Windows.

### Cost study

The Department of Financial Management Control in our hospital receives information on activity and financial information on all the hospital’s subsystems. Here is where data on hospital activity and data on the costs derived from this hospital activity are sent. This model is used to create a system of imputation that calculates the cost of each patient episode, not by making an estimate according to the patient’s DRG weight, but by totaling the cost of all the intermediate products the patient receives.

This was how the cost of each inappropriate stay of the sample patients was calculated. Taking into account the rate of inappropriateness of each Medical Service and the mean cost of hospitalization, we extrapolated these data to calculate the approximate cost of inappropriate stays in our hospital in 2005 and 2007. To make the financial calculation we updated the cost of each patient by applying the Consumer Price Index to the level of December 2007.

## Results

### Descriptive study

A sample of 1450 histories was studied (725 belonging to the control group and 725 to the intervention group). The admission protocol was applied to the 1450 histories and the inappropriate stay protocol to 2675 stays.

The patient sample was made up of 813 (56.1%) males and 637 (43.9%) females, with a mean age of 62 + 19 years (range: 11–97).

### Type of admission

1,169 patients (80.6%) had emergency admissions and 281 patients (19.4%) were programmed.

### Distribution by medical services

The Service with the highest percentage of histories studied was Internal Medicine with 425 (29.3%), followed by General Surgery with 258 (17.8%), Other Medical with 183 (12.6%), Cardiology with 154 (10.6%), Orthopedic with 162 (11.2%), Hematology with 105 (7.2%), Other Surgery with 95 (6.6%) and Infectious Diseases with 68 (4.7%) (Figure 1).

### Time of admission

The distribution of cases by day of the week on which hospital admission occurred ranged from 17.9% of admissions on a Monday to 8.3% of admissions on a Saturday. The month with the highest rate of admissions was June (10.5%) and with the lowest August (5.7%). Nineteen point six percent of the patients were admitted during the holiday period and 80.4% during the normal working period.

### Comparison of groups

Both of the groups (control and intervention) were homogeneous, with no significant differences in variables: age, distribution by sex, distribution by medical service, month and day of admission (Table 2).

**Table 2:** Comparison of independent variables in the control group (CG) and intervention group (IG)

		**CG**	**IG**	**p**
Type of Service				
	Medical	468 (64.6)	468 (64.6)	1
	Surgical	257 (35.4)	257 (35.4)	
Day of Admission				0.662
	Monday	130 (7.9)	134 (18.5)	
	Tuesday	127 (17.5)	116 (16.0)	
	Wednesday	116 (16.0)	121 (16.7)	
	Thursday	94 (13.0)	110 (15.2)	
	Friday	101 (13.9)	92 (12.7)	
	Saturday	60 (8.3)	74 (10.2)	
	Sunday	97 (13.4)	78 (10.8)	
Weekend Admission				0.798
	YES	157 (21.7)	153 (21.1)	
	NO	568 (78.3)	57 (78.9)	
Sex				0.711
	Male	410 (56.6)	403 (55.6)	
	Female	315 (43.4)	322 (44.4)	
Type of Admission				0.790
	Programmed	143 (19.7)	138 (19.0)	
	Emergency	582 (80.3)	587 (81.0)	
Age		62.6 ± 19.4	61.8 ± 20.1	0.436
Number of Comorbidities				0.616
	1	244 (57.7)	225 (54.6)	
	2	103 (24.3)	115 (27.9)	
	3	50 (11.8)	46 (11.2)	
	4	23 (5.4)	21 (5.1)	
	5	3 (0.7)		5 (1.2)
Weight		1.863 ± 1.633	1.857 ± 2.096	0.953
Total Length of Stay		9.3 ± 8.2	8.0 ± 6.9	<0.001
Outlying Admission		257 (35.4)	175 (24.1)	<0.001

The total length of stay in the control group was 9.3 ± 8.2 days and in the intervention group 8.0 ± 6.9 days (p < 0.001) (Table 2).

Outlying patients (those admitted outside their nursing unit) accounted for 257 (35.4%) of the control group and 175 (24.1%) of the intervention group (p <0.001) (Table 2).

The rate of occupancy on the day the sample patients were admitted was 85.4 ± 6.2 in the control group and 84.7 ± 4.2 in the intervention group (p = 0.022). The number of admissions per day was 44 ± 12 in the control group and 47 ± 13 in the intervention group (p < 0.001) (Table 2).

There were no significant differences in the percentage of patients with emergency or programmed admissions. Both groups were homogeneous for DRG distribution, ICD-9 group diagnosis classification and comorbidities. Patient weight was 1.8634 ± 1.6335 in the control group and 1.8576 ± 2.0959 in the intervention group (p = 0.953) (Table 2).

### Comparison of inappropriate admissions

In the control group, when comparing medical and surgical services, we see that inappropriate admission is 10.9% for medical services and 1.3% for surgical services (p < 0.001) (Table 3). There are also significant differences among the different clinical services. As for age and sex (Table 3), older patients had fewer inappropriate admissions than younger patients (p = 0.01) but there are no significant differences when comparing the results of inappropriate admission by gender (male/female). Emergency admissions had a somewhat higher inappropriateness (7.9%) than programmed admissions (5.6%) (p = 0.346). There were no differences in inappropriateness according to the hospital occupancy rate on the day of admission.

**Table 3:** Variables related to inappropriateness of admission Control group

		**Appropriate**	**Inappropriate**	**p**
**Type of Service**				**<0.001**
	Medical	417 (89.1)	51 (10.9)	
	Surgical	254 (98.8)	2 (1.3)	
**Weekend**				**0.731**
	YES	147 (93.6)	10 (6.4)	
	NO	524 (91.0)	44 (7.7)	
**Holidays**				**0.202**
	YES	135 (95.1)	7 (4.9)	
	NO	536 (91.9)	47 (8.1)	
**Sex**				**0.895**
	Male	379 (92.4)	31 (7.6)	
	Female	292 (92.7)	23 (7.3)	
**Type of Admission**				**0.346**
	Programmed	135 (94.4)	8 (5.6)	
	Emergency	536 (92.1)	46 (7.9)	
**Origin**				**0.315**
	Emergencies	423 (91.6)	39 (8.4)	
	Consultation	62 (91.2)	6 (8.8)	
	Home	88 (96.7)	3 (3.3)	
	Other Centre	98 (94.2)	6 (5.8)	
**Age**		63.1 ± 19.4	56.9 ± 17.9	**0.010**
**Rate of Occupancy**		85.4 ± 6.2	85.1 ± 6.9	**0.804**
**Number of Admissions**		44.2 ± 12.4	45.9 ± 10.9	**0.352**

Control group.

**Figure 2:**
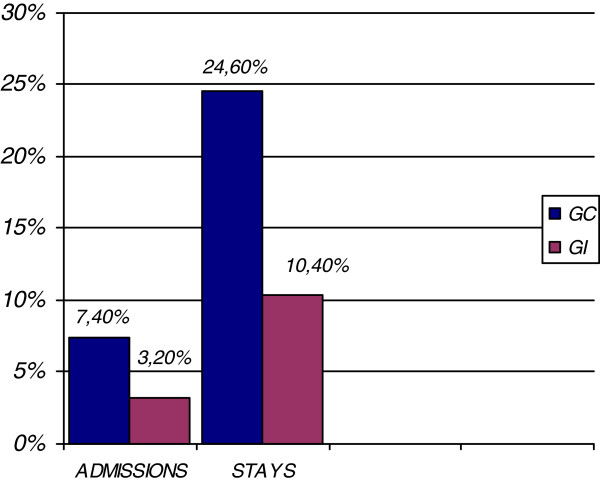
Comparison of inappropriate admissions and stays and cost of inappropriateness of the sample in the Control group (CG) and Intervention group (IG).

Comparing control and intervention groups, inappropriateness of admission in the control group was 7.4% (54 patients), whereas in the intervention group it was significantly reduced to 3.2% (23 patients) (p < 0.001) (Figure 2).

The cause of most inappropriate admissions (“diagnostic tests and/or treatments that can be performed in outpatients”) was revealed by 46 (6.3%) patients in the control group and 22 patients (3.0%) in the intervention group, a reduction that is not statistically significant.

Inappropriateness was reduced in most of the medical services and more significantly in those that had a higher rate of inappropriateness in the control group. Internal medicine had a 9.4% rate of inappropriateness in the control group and 3.7% in the intervention group (p = 0.019); cardiology had 15% in the control group and 3% in the intervention group (p = 0.087); and neurology had 24.4% in the control group and 4.8% in the intervention group (p = 0.025) (Table 4).

**Table 4:** **Comparison of inappropriate stays by medical service Control Group (CG) *vs *Intervention Group (IG)**

	**CG (n: 725)**	**IG (n:725)**	**p**
Internal medicine	20 (9.4)	8 (3.7)	0.019
Cardiology	12 (15.0)	3 (4.1)	0.087
Infectious Diseases	1 (2.9)	1 (2.9)	1,000
Oncohaematology	3 (5.8)	4 (7.5)	0.979
Neurology	10 (24.4)	2 (4.8)	0.025
General Surgery	0	2 (1.6)	0.498
Orthopedic	3 (3.7)	0	0.245
Pneumology	1 (4.1)	0	1.000
Digestive Diseases	3 (15.7)	3 (15.0)	1.000
Rheumatology	1 (25.0)	0	1.000
Ophthalmology	0		0
Otorhinolaryngology	0	0	
Urology	0	0	
Endocrinology	0		0

Control Group (CG) Intervention Group (IG).

### Comparison of inappropriate stays

In the control group the surgical patients had an inappropriate stay rate of 18.1% and the medical patients 28.3% (p < 0.001). With regard to age, younger patients had a greater likelihood of inappropriate stay than older patients (p = 0.030). As far as DRGs are concerned, and differentiating between medical and surgical DRGs, the inappropriate stay rate was 34.1% for medical and 25.8% for surgical (p = 0.025). There was a higher rate of appropriate stays when the hospital bed occupancy rate was higher (85.5% ± 6.6% *versus* 84.5% ± 6.5%; p = 0.010) (Table 5).

**Table 5:** Inappropriate stays in the control group Related variables

	**Appropriate (n:1021) (%)**	**Inappropriate (n:334) (%)**	**p**	
**Outlying**			**0.001**	
YES	343 (70.3)	145 (29.7)		
NO	678 (78.2)	189 (21.8)		
**Holidays**			**0.534**	
YES	196 (76.9)	59 (23.1)		
NO	825 (75.0)	275 (25.0)		
**Weekend**			**0.083**	
YES	306 (72.3)	117 (27.7)		
NO	715 (76.7)	217 (23.3)		
**Total Length of Stay**	10.0 ± 8.9	8.8 ± 6.3	**0.010**	
**Sex**			**0.290**	
Male	584 (76.4)	180 (23.6)		
Female	437 (73.9)	154 (26.1)		
**Type of Admission**			**0.037**	
Programmed	212 (80.3)	52 (19.7)		
Emergency	809 (74.2)	282 (25.8)		
**Appropriateness of Admission**			**<0.001**	
Appropriate	995 (79.3)	259 (20.7)		
Inappropriate	26 (25.7)	75 (74.3)		
**Age**	63.7 ± 19.5	61.2 ± 17.5	**0.030**	
**Rate of Occupancy**	85.5 ± 6.6	84.5 ± 6.5	**0.010**	

Related variables.

Comparing control and intervention groups, the percentage of inappropriate stays was 24.6% (334 patients) in the control group and 10.4% (137 patients) in the intervention group (p < 0.001) (Figure 2).

The causes justifying most inappropriate stays were reduced significantly; “patients with a diagnostic or therapeutic procedure that can be performed on an outpatient basis” was observed in 188 patients (13.8%) from the control group and 56 patients (4.2%) from the intervention group; “patients pending results of diagnostic tests” was seen in 155 patients (11.4%) from the control group and 85 patients (6.4%) from the intervention group; and “patients admitted as a result of conservative attitude of doctor” was reduced from 116 patients (8.5%) in the control group to 26 patients (1.9%) in the intervention group.

The medical services with the highest rate of inappropriateness in the control group were those that reduced this inappropriateness most significantly in the intervention group: internal medicine went from a 22.7% inappropriateness to 13.8% (p < 0.001); cardiology went from 48.3% to 16.3% (p < 0.001); infectious diseases from 17.4% to 6.9% (p = 0.132); neurology from 53.3% to 26.5% (p < 0.001); orthopedic from 27.1% to 2.7% (p < 0.001); and digestive diseases from 50.0% to 23.6% (p = 0.035) (Table 6).

**Table 6:** **Comparison of inappropriate stays by medical service Control Group (CG) *vs *Intervention Group (IG)**

	**CG (n:1355)**	**IG (n:1320)**	**P**
Internal Medicine	89 (22.7)	52 (13.8)	<0.001
Cardiology	3 (48.3)	24 (16.3)	<0.001
Infectious Diseases	1 (17.4)	4 (6.9)	0.137
Oncohaematology	(4.8)	3 (3.1)	0.772
Neurology	0 (53.3)	22 (26.5)	<0.001
General Surgery	31 (13.1)	10 (4.2)	<0.001
Orthopedic	43 (27.1)	4 (2.7)	<0.001
Pneumology	(4.2)	3 (6.5)	0.677
Digestive Diseases	18 (50.0)	9 (23.6)	0.035
Rheumatology	6 (75.0)	0	0.466
Ophthalmology	4 (100)	3 (75.0)	1.000
Otorhinolaryngology	0	3 (15.8)	0.084
Urology	10 (16.1)	0	0.001
Endocrinology	2 (40.0)	0	0.181

Control Group (CG) Intervention Group (IG).

### Cost of inappropriateness

The cost of the days considered inappropriate in the study sample, taking into account the mean cost per patient, clinical service and day, was 147,044 euros in the control group and 66,462 euros in the intervention group.

Extrapolating the results to the rate of inappropriateness of each clinical service out of the total of admissions and hospital stays we calculated the approximate minimum cost of inappropriate admissions and stays in 2005 and 2007 per Service and for the Hospital as a whole, which amounts to 2,125,638 euros in 2005 (control group) and 960,761 euros in 2007 (intervention group).

## Discussion

The increase in health costs has become a serious concern in our environment. The rational use of hospital resources is a matter which with time is becoming more important, since health resources are growing at a lower rate than the needs of the population. The resources spent as a result of prolonged hospital stays are one of the components than can currently be acted upon. Reducing inappropriate admissions and stays implies: lower costs due to a reduction in unnecessary hospital use; improved health care quality due to a reduction in inappropriate procedures, iatrogenic diseases and nosocomial infections; and, most importantly, better accessibility to health care, with hospital care reserved for those who really need it.

### The AEP questionnaire

The initial studies by Gertman and Restuccia to develop the AEP go back more than 25 years [3]; around the same time are the recommendations by Donabedian [28] to avoid an absolute reduction in unnecessary hospital use, with the suggestion that this would also imply a problem of inappropriateness due to underuse.

The AEP is a moderately valid and highly reliable instrument [8]. All the studies which have evaluated the AEP show that it has a great capacity for reproducing reliable results in different hospital environments and with different reviewer profiles. The degree of concordance attained in our study by the three assessors following a short training period is very high, with kappa values of 0.77 for appropriate stays and 0.31 for appropriate admissions. As mentioned previously, it should be stressed by virtue of the mathematical formula for calculating the kappa index that when the concordance among assessors is very high, as occurred in the case of appropriate admissions, the kappa value is paradoxically very low [27]. The AEP shows greater concordance with any clinical reviewer than with any pair of clinical reviewers between them, using implicit criteria [5].

The AEP has had various applications, corresponding usually to inappropriateness studies in specific clinical services [11-17]. Only a few studies have conducted a global inappropriateness study on a hospital as a whole [18,19].

In recent years a gradual evolution has been observed from merely descriptive studies to interventions to modify clinical practice [21], since a study on inappropriate hospital use without the intention to reduce inappropriateness would make no sense.

Most interventions for improvement have been implemented using feedback to physicians [22-25], who have seen how this feedback has been effective for reducing both inappropriate stays and total length of stay. The main limitation of these studies is that the effect of this measure usually finishes once the study is concluded.

Interventions must be based on previous identification of the factors influencing this inappropriateness, analysis of the causes and a joint proposal of measures to be adopted by hospital management and doctors [22]. Most previously described interventions use intervention measures without prior analysis of a series [26,29], which explains why expected improvements are not always made. Antón [26] obtains a significant decrease in inappropriate admissions but not in stays and Moya [24] obtains a reduction in inappropriateness that can be attributed to doctors’ attitudes but fails to properly reduce inappropriate stays.

Analysis of the causes of inappropriateness in our study led to two groups of measures being proposed: one aimed at the hospital’s doctors based fundamentally on feedback and one with joint participation of the hospital management and the doctors based on improving patient circuits and relocating the offer of preferential services.

The first identified cause of inappropriate admission or stay was “patient awaiting tests that can be done on an outpatient basis”. This resulted in the proposed creation of a special outpatient agenda in the radiodiagnosis service for patients from emergencies or for those newly diagnosed with oncological disease in order to avoid their being admitted. The number of patients inappropriately admitted for this cause was thus reduced significantly from 46 (6.3%) to 22 patients (3%). Likewise there was a reduction in the two causes of inappropriate stay related to a delay in diagnostic tests: “patient awaiting tests” was observed in 188 patients (13.8%) in the control group compared to 56 patients (4.2%) in the intervention group (p < 0.001) and “patient awaiting test results” in 155 patients (11.4%) in the control group *versus* 85 patients (6.4%) in the intervention group (p < 0.001).

A reduction in the third most frequent cause of inappropriate stay (“conservative attitude of doctor”) was attempted by using feedback to physicians. Educational sessions were programmed by clinical services, in which the use of a discharge pre-report was proposed and the evaluation results of the control group and the measures adopted to reduce the inappropriateness were reported. Inappropriateness due to this cause fell from 116 patients (8.5%) in the control group to 26 patients (1.9%) in the intervention group.

Location of patients outside their nursing unit (“outlying”) has been considered by few authors [11]. The condition of outlying patient in our study was significantly associated with a greater inappropriateness of stay. The adopted measures reduced the number of outlying patients from 257 (35.4%) in the control group to 175 (24.1%) in the intervention group.

Although the main objective of reducing inappropriateness is to reserve the use of hospital resources for patients who really need them, the financial saving implied by the reduction in inappropriateness in our hospital would be 1,164,877 euros. This estimated figure should make management members and medical staff think about their share of the responsibility for the adequate use of health resources.

### Biases and limitations of the study

The main limitation of the study lies in its design. The effectiveness of an intervention should be tested with a controlled randomized assay on 2 parallel groups. We conducted our study with a quasi-experimental pre- and post-test, as we thought that the logistic difficulties of intervening in some patients and not others, receiving attention at different levels of hospital care, would make it difficult for the study to be carried out. Secondly, many of our data were analyzed retrospectively, with the inherent difficulty of ensuring the validity of the data collected. Thirdly, we acknowledge that despite our intervention there are other factors (administrative, managerial, etc.) that might have influenced the results obtained. Finally, the collection of data by 3 assessors may condition a variability that can hamper the credibility of the data obtained; we therefore studied inter-observer concordance and obtained a good concordance. Despite these limitations we believe that they do not invalidate our results and conclusions.

## Conclusions

A reduction in inappropriate admissions and stays can be achieved, but to ensure the success of any intervention the measures adopted must be based on an analysis of the data and causes of inappropriateness in each centre.

## Competing interest

The authors declare that they have no competing interest.

## Authors' contribution

VS conceived the study and participated in the design to draft the manuscript AC participated in the design of the study and performed the statistical analysis, giving final approval of the version to be published BF made substantial contributions to the conception and design of the manuscript. AM revised the manuscript critically for important intellectual content. MC participated in the sequence alignment and drafted the manuscript and gave final approval of the version to be published. JA participated in the sequence alignment and drafted the manuscript and gave final approval of the version to be published. All authors read and approved the final manuscript.

## Pre-publication history

The pre-publication history for this paper can be accessed here:

http://www.biomedcentral.com/1472-6963/12/361/prepub
